# Piperine synergistically enhances the effect of temozolomide against temozolomide-resistant human glioma cell lines

**DOI:** 10.1080/21655979.2020.1794100

**Published:** 2020-07-21

**Authors:** Somi Jeong, Seunghwa Jung, Gyun-Seok Park, Juhyun Shin, Jae-Wook Oh

**Affiliations:** a Department of Stem Cell and Regenerative Biotechnology, Konkuk Institute of Technology, Konkuk University, Seoul, Korea; b Department of Bio-resources and Food Science, Konkuk University, Seoul, Korea

**Keywords:** Piperine, temozolomide, glioblastoma multiforme, inhibition of cell growth, apoptosis

## Abstract

Temozolomide (TMZ) is an alkylating chemotherapy agent used in the clinical treatment of glioblastoma multiforme (GBM) patients. Piperine (PIP) is a naturally occurring pungent nitrogenous substance present in the fruits of peppers. We investigated the anti-cancer efficacies of PIP alone and in combination with TMZ in GBM cellsusingparameters such as cell proliferation, cellular apoptosis,caspase-8/-9/-3 activities, cell cycle kinetics, wound-healing ability, and loss of mitochondrial membrane potential (MMP). Treatment with PIP and alow concentration of PIP-TMZ, inhibited cell growth, similar to TMZ.PIP-TMZ promoted apoptosis by activation of caspase-8/-9/-3, MMP loss, and inhibition of *in vitro* wound-healing motility. Reverse transcription polymerase chain reaction analysis showed significant inhibition of *Cyclin-dependent kinases* (*CDK)4/6−cyclin D* and *CDK2−cyclin-E* expression upon treatment with a low concentration PIP-TMZ, suggesting an S to G1 arrest. Our findings provide insight into the apoptotic potential of the combination of a low concentration of PIP-TMZ, though further *in vivo* study will be needed for its validation.

## Introduction

Glioblastoma multiforme (GBM) is one of the most common types of malignant brain tumors [1]. Due to its high invasiveness and heterogeneity, gliomas exhibit resistance to traditional treatments, including surgery, irradiation, and chemotherapy [2]. The average life span for patients diagnosed with glioblastoma has not changed substantively over the previous years and remains at 8‒12 months [3]. Thus, there is an urgent need to develop novel therapy strategies.

Temozolomide (TMZ), an alkylating agent that contains an imidazole tetrazine ring, isone of the most effective drugs for clinical GBM treatment [4]. TMZ easily penetrates the blood-brain barrier further delaying the recurrence of glioma [5], and triggers apoptosis by inducing DNA lesion O^6^-methylguanine and a change in the role of p53 [6]. However, clinical data demonstrates that patients require a high dose of TMZ, which presents obvious toxic side effects [7]. Based on this knowledge, the efficacy of many targeted therapies in combination with TMZ has been tested [8,9]. Unfortunately, to date these efforts have not led to any successful therapies, so improving treatment effectiveness of TMZ remains a priority.

Piperine (PIP) is the principal biological component in black pepper [10]. PIP possesses multifunctional pharmacological properties such as anti-inflammatory, antioxidant, antidiarrheal, hypolipidemic, hepato-protective, anti-mutagenic, antimicrobial, and anti-carcinogenic activities [11–13]. PIP has also inhibited the growth of breast carcinoma by targeting the renewal of cancer stem cells [14]. Furthermore, a recent study has revealed that PIP inhibits the proliferation of HeLa cells by caspase-mediated apoptosis [15]. Despite these indirect effects of PIP, little is known about the anti-cancer effect of PIP on human GBM cells.

In the present study, we focused on examining the anti-cancer potential of PIP to increase the effect of TMZ by measuring potent apoptosis markers such as cell proliferation, caspase-8/-9/-3 activation, cell cycle checkpoints,*CDK4/6−cyclin D* and *CDK2−cyclin-E* gene expression levels, JNK and p38 MAPK activation, delayed *in vitro* motility for wound healing, and mitochondrial membrane potential (MMP) reduction in the human GBM cell lines.

## Materials and methods

### Cell culture

The human U251-MG glioma cell line was obtained from Dr Benveniste EN (University of Alabama at Birmingham, Birmingham, USA). The T98G human astroglioma cell lines from American Type Culture Collection (ATCC CRL-1690, USA) were purchased. Cells were determined to be mycoplasma free, based on Biomax’s mycoplasma PCR analysis kit (Biomax Inc., Daejeon, Korea). For these cells, Minimum Essential Medium Eagle (HyClone) with 10% fetal bovine serum (FBS) (HyClone), supplemented with 2 mM L-glutamine, 100 U/ml penicillin, 100 µg/ml streptomycin, and 1 mM sodium pyruvate (Welgene) were used.

### Cell viability assay

Cells (2 × 10^4^) were plated in each well of a 96-well microplate (SPL). The following day each cell was treated with the specified concentration of PIP, TMZ (Sigma-Aldrich), or low concentration PIP-TMZ for 24‒72 h. Then, 10 µl WST-1 solution (0.5 mg/ml) was added in each well and incubated in the dark for 1 h. Cell viability was measured using a colorimetric assay with 2-(4-iodophenyl)-3-(4-nitrophenyl)-5-(2,4-disulfophenyl)-2 H-tetrazolium monosodium salt (WST-1) (Roche Applied Science). The absorbance was determined at 450 nm and read with Gen5 software (Bio-Tek). Untreated cells were set as 100 percentage viability and the relative cell viability was calculated as the mean of percentage for each triplicate.

### Morphological observation

Cells (2 × 10^4^) were plated in a six-well plate and the next day the cells were treated with the indicated concentration of PIP, TMZ, or PIP-TMZ in combination; then, morphological changes were verified with imaging at 24 h under an inverted phase-contrast microscope (Motic)

### Cell cycle analysis and flow cytometric analysis

Cells were seeded into six-well plates at 2 × 10^5^ cells per well and incubated for 24 h under the treatment conditions of PIP, TMZ, and PIP-TMZ in combination. Then, adherent and floating cells were fixed in a PBS-methanol solution and maintained at 4°C for at least 18 h. After wash with PBS, cell pellets were stained with a fluorescent probe Propidium Iodide (PI) (Thermo Fisher Scientific) containing PBS, 20 µg/ml PI, and 40 µg/ml DNase-free RNase A for 30 min in the dark. DNA fluorescence of stained cells was analyzed by a NovoCyte 1000 benchtop flow cytometer (ACEA biosciences). The DNA histograms were evaluated using Modfit software to calculate the percentage of cells in various phases of the cell cycle.

The status of apoptosis was evaluated using FITC-Annexin V apoptosis detection method (BD Pharmingen). The cells seeded into six-well plates at 2 × 10^5^ cells/well were treated with PIP, TMZ, and PIP-TMZ and incubated for 24 h. Then, cells were washed twice and resuspended in 1 ml of Annexin V binding buffer at a concentration of 1 × 10^6^ cells/ml. 100 µl of the resuspended solution (1 × 10^5^ cells) was transferred to a 1.5 ml microtube and 5 µl of 1× FITC-Annexin V and 5 µl PI were added. After incubation for 20 min, 400 µl of 1× Annexin V binding buffer was added to each sample. Finally, cells were analyzed by flow cytometry using the NovoCyte 1000 and visualized by NovoExpress.

### Western blot

Prepared cells were lysed in lysis buffer (1% NP-40, 0.5% sodium deoxycholate, 0.1% SDS, 50 mM Tris-Cl, 0.02% sodium azide, and 150 mM NaCl, supplemented with 1 mM PMSF, 2 µg/mL aprotinin, 1 µg/mL leupeptin, 1 µg/mL pepstatin A, 2 mM sodium fluoride, and 1 mM sodium orthovanadate). Denatured lysates were loaded into the wells with a 10% sodium dodecyl sulfate-polyacrylamide gel electrophoresis (SDS-PAGE) and transferred onto a polyvinyl difluoride (PVDF) membrane. After blocking for 1 h with 5% nonfat milk, membranes were incubated overnight at 4°C with cognate Ab [Anti-phospho-ERK/-JNK/-p38 MAPKs Ab; anti-caspase 8/-9 Ab; anti-actin Ab (Santa Cruz) and caspase-3 Ab (CST)]. After incubating, membranes were incubated with Horseradish peroxidase (HRP)-conjugated goat anti-mouse IgG Ab or goat anti-rabbit IgG Ab (Jackson ImmunoResearch Lab) for 2 h. Bands were visualized by enhanced chemiluminescence (Bio-Rad).

### RT-PCR

Total RNA was extracted from prepared cells using TRIzol reagent (Ambion). First-strand DNA synthesis was conducted with oligo (dT) and AccuPower® CycleScript RT PreMix kit (BIONEER), followed by PCR with 2× Thumb Taq PCR PreMix (BioFACT). The thermocycler profiles for cell cycle-related genes were as follows: one cycle at 95°C for 2 min, 30 cycles of 95°C for 20 s, 55 ~ 58°C for 40 s, and 72°C for 1 min; ending with one cycle at 72°C for 5 min. The amplification program for *GAPDH* was the same, but will the cycle number reduced to 25. PCR products were detected using a 2% agarose gel with ethidium bromide and analyzed using ChemiImager 5500 (Alpha Innotech). Primers used in this study are listed in Table 1. Values for each mRNA product were normalized to *GAPDH* mRNA levels for each experimental condition.

**Table 1.** Primers used in this study.

Gene name	Accession number	Primer sequences (5ʹ to 3ʹ)
Cyclin D1	NM_053056.2	Forward: TGGATGCTGGAGGTCTGCGAGGAA
		Reward: GGCTTCGATCTGCTCCTGGCAGGC
Cyclin E	NM_001238.4	Forward: AGTTCTCGGCTCGCTCCAGGAAGA
		Reward: TCTTGTGTCGCCATATACCGGTCA
Cdk2	NM_001290230.2	Forward: GCTTTCTGCCATTCTCATCG
		Reward: GTCCCCAGAGTCCGAAAGAT
Cdk4	NM_000075.4	Forward: ACGGGTGTAAGTGCCATCTG
		Reward: TGGTGTCGGTGCCTATGGGA
Cdk6	NM_001145306.1	Forward: CGAATGCGTGGCGGAGATC
		Reward: CCACTGAGGTTAGAGCCATC
GAPDH	NM_001256799.3	Forward: GTCTTCACCACCATGGAGAA
		Reward: CGTTCAGCTCTGGGATGACC

### In‑vitro wound-healing assay

Cells (2 × 10^5^ per well) were plated in a six-well plate (SPL) and incubated overnight. A liner scratch wound was created after 24 h of 1% FBS containing medium condition with the help of a sterile 0.2 ml yellow tip. Cell debris was cleared by washing twice with PBS, and subsequently, PIP, TMZ, and PIP-TMZ treatments were completed. The wound was analyzed and photographed at 24 h under an inverted phase-contrast microscope and imaged by Motic Image Plus 2.0 ML (Motic).

### Mitochondrial membrane potential (MMP) assessment

The decrease in mitochondrial membrane potential (ΔΨm) of cells after a 24 h exposure of PIP, TMZ, and PIP-TMZ was evaluated by potentiometric fluorescent dye (5,5ʹ,6,6ʹ-tetrachloro-1,1ʹ,3,3ʹ-tetraethyl benzimidazolecarbocyanine iodide (JC-1) (Enzo Life Sciences). The patterns of mitochondrial depolarization were observed with the help of a FACs analysis using the NovoCyte 1000 and visualized by NovoExpress, by pairing the red and green fluorescent counts for cell quantification analysis.

### Statistical analysis

Standard deviation (± SD) was measured for replicates of each duplicated independent experiment. Statistical analyses were performed using the IBM statistical package for the social sciences (SPSS) for Windows version 24.0 (SPSS Inc., Chicago, IL, USA). A one-way analysis of variance (ANOVA) was used to determine overall significant differences. For multiple comparison evaluation of statistical significances, a two-tailed Student’s *t*-test was used (*p < 0.05 and **p < 0.01).

## Results

**Figure 1.**
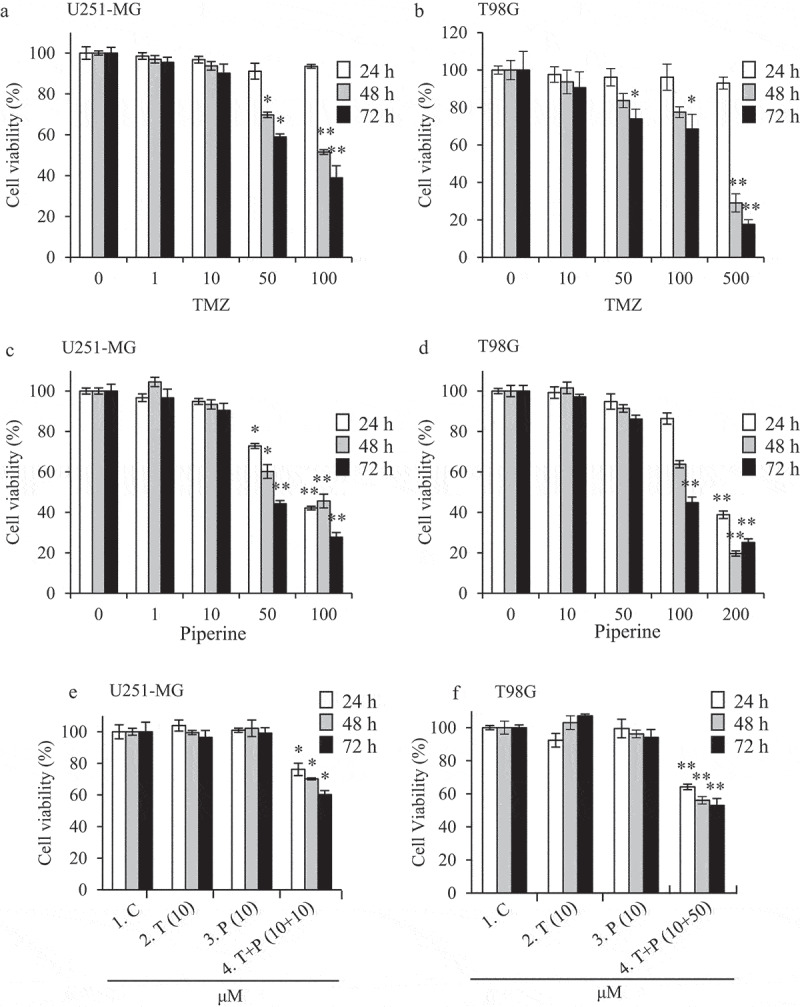
Effect of PIP and TMZ alone or in combination on cell viability of human GBM cell lines (a and b).

We evaluated the effects of PIP alone or in combination with TMZ on the viability of glioma cells. As shown in Figure 1(a,b), cell viability was not reduced in response to increasing concentrations (0‒500 µM) of TMZ for 24 h, indicating these cells have a strong resistance to TMZ. However, cells treated with TMZ (50‒500 µM) for 48‒72 h showed less viability (~ 60‒80% inhibition) compared to control. Interestingly, as shown in Figure 1(c,d), cell viability decreased in response to increasing concentrations (0‒200 µM) of PIP for 24‒72 h. Strikingly, the cell viability decreased significantly after treatment with combinations of low levels of PIP (10 or 50 µM) and TMZ (10 µM) for 24‒72 h compared to alone, suggesting that PIP has an anti-cancer effect in GBM cells as well as synergistically enhances the anti-cancer effect of TMZ (Figure 1(e,f)).

Effect of TMZ (0, 1, 10, 50, 100, or 500 µM) on cell viability of U251-MG and T98G at 24, 48, and 72 h (c and d). Effect of PIP (0, 1, 10, 50, 100, or 200 µM) on cell viability of U251-MG and T98G at 24, 48, and 72 h (e and f). Synergistic effect of low concentration PIP-TMZ (10 + 10/50 + 10 µM) on cell viability of U251-MG and T98G at 24, 48, and 72 h. Data shown are mean ± SD of three similar experiments (*p < 0.05 and **p < 0.01).

**Figure 2.**
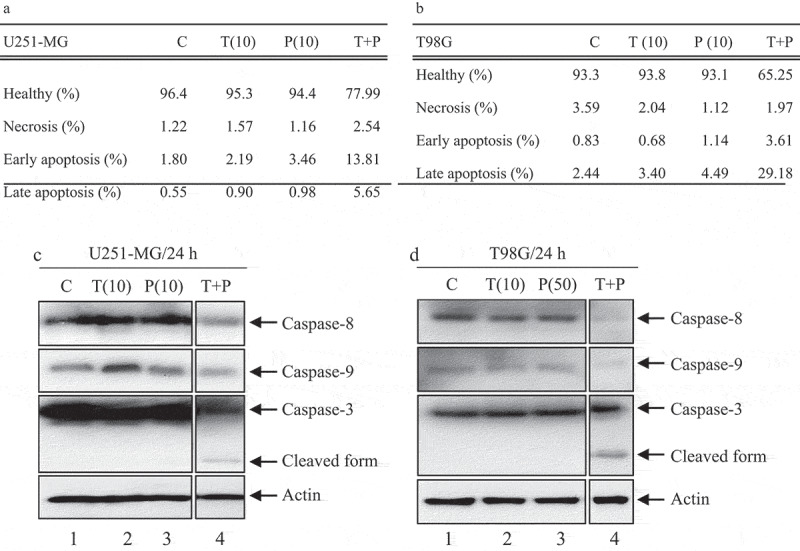
Effect of PIP and TMZ alone or in combination on apoptosis of human glioma cells.

Next, we quantified the number of apoptotic cells by flow cytometry. As shown in Figure 2(a,b), as expected the percentages of apoptotic cells in U251-MG cells (A) treated with low concentration PIP (10 µM) or TMZ (10 µM) were 4.44% and 3.09%, respectively. In T98G cells (B) treated with low concentration PIP (50 µM) or TMZ (10 µM) were 5.63% and 4.08%, respectively, compared to controls (2.35% and 3.23%), suggesting that low concentration of PIP and TMZ alone failed to induce apoptosis. However, after low concentrationPIP-TMZ treatment, the percentages of apoptotic cells significantly increased. The total apoptosis rate of U251-MG cells in low PIP-TMZ (10 + 10 µM) was 19.46% (A), consisting of 13.81% in early apoptosis and 5.65% in late apoptosis. The total apoptosis rate of T98G in low PIP-TMZ (10 + 50 µM) was 32.79% (B), consisting of 3.61% in early apoptosis and 29.18% in late apoptosis, suggesting that low PIP-TMZ combination leads to synergistic cytotoxic effect of the GBM cells. To investigate the apoptotic molecular mechanisms in response to a low PIP-TMZ, western blot was carried out. As shown in Figure 2(c,d), no obvious bands of active caspase-8/-9/-3 could be observed in cells treated with a low concentration of PIP (10 or 50 µM) or of TMZ (10 µM) alone. However, low PIP-TMZ [10 + 10 µM (C) or 50 + 10 µM (D)]treatmentfor 24 h significantly decreased uncleaved caspase-8/-9 expression. In the case of caspase-3, the band of cleaved form was weak in U251-MG cell (C), but the clear band was detected in T98G cell (D), therefore suggesting an increase of cleaved (active) caspases activity upon treatment (*lane 4/each*). These results suggest that low PIP-TMZ accelerates the activation of the apoptosis pathway in TMZ-resistant GBM cells.

PIP (10/50 µM) and TMZ (10 µM) alone or in combination were treated on U251-MG (a) and T98G (b) cells for 24 h, then flow cytometry was performed to analyze the percentage of apoptotic cells. Data presented is representative of three experiments. The U251-MG (c) and T98G (d) cells were treated with PIP (10/50 µM) alone or in combination with TMZ (10 µM) for 24 h. The proteins in cell lysates were determined by western blot. Actin was used as a loading control. The data show the mean ± SD of three independent experiments.

**Figure 3.**
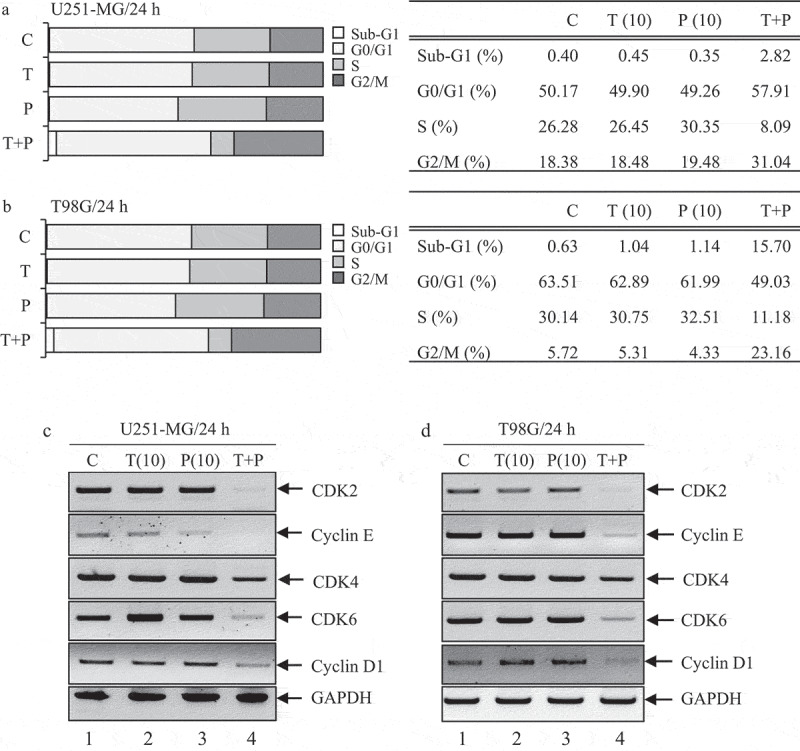
The analysis of cell cycle and expression levels of *Cdks/Cyclins* after stimulation with PIP and TMZ alone, or in combination on GBM cells.

We analyzed the synergistic effect of low PIP-TMZ on the cell cycle by flow cytometry. As shown in Figure 3(a), the percentage of U251-MG cells in G0/G1 phase in low concentration PIP (10 µM) and TMZ (10 µM) alone was found to be 49.26% and 49.90%, respectively, and in low PIP-TMZ (10 + 10 µM) the cell cycle checkpoint showed a slight increase to 57.91%. While the subG1 phase (an indication of apoptosis) was found to be 0.35% and 0.45% for low concentration PIP (10 µM) and TMZ (10 µM); however, it increased markedly to 2.82% for low PIP-TMZ (10 + 10 µM). Interestingly, the cell population in S-phase declined steeply in low PIP-TMZ (8.09%), compared to PIP and TMZ alone (30.35% and 26.35%, respectively). Comparable results were seen in T98G cells (Figure 3(b)). These results show that low PIP-TMZ synergistically induces cellular apoptosis followed by cell cycle arrest at the G1/S checkpoint and by increasing in the subG1 phase of the cell cycle in GBM , we performed RT-PCR to determine the effect of low PIP-TMZ on cell cycle-related genes that control progression through the G1 phase [15]. As shown in Figure 3(c,d), high constitutive CDKs/Cyclins mRNA expression was detected (*lane 1*), while low PIP/TMZ-treated U251-MG and T98G cells exhibited a significant decrease in their expression of *cyclin D1, cyclin E, Cdk2, Cdk4*, and *Cdk6* genes (*lane 4/each*), compared to the PIP and TMZ-only treatments (*lane 2‒3*).

U251-MG (a) and T98G cells (b) (3 × 10^5^ cells) were incubated in 1.5 ml serum-free media with low concentrationPIP (10/50 µM) and low concentration TMZ (10 µM) alone, or in combination. Cells were stained with PI/Annexin V-FITC and analyzed by flow cytometry. Data presented is representative of three experiments. U251-MG (c) and T98G (d) cells were incubated for 24 h in the presence of low concentration PIP (10/50 µM) and low concentration TMZ (10 µM) alone or in combination. Total RNA was extracted and RT-PCR was performed. Expression levels of *Cdk2/4/6* and *Cyclin D1/E* genes were analyzed. *GAPDH* was used as a loading control. Results from a representative experiment are shown.

**Figure 4.**
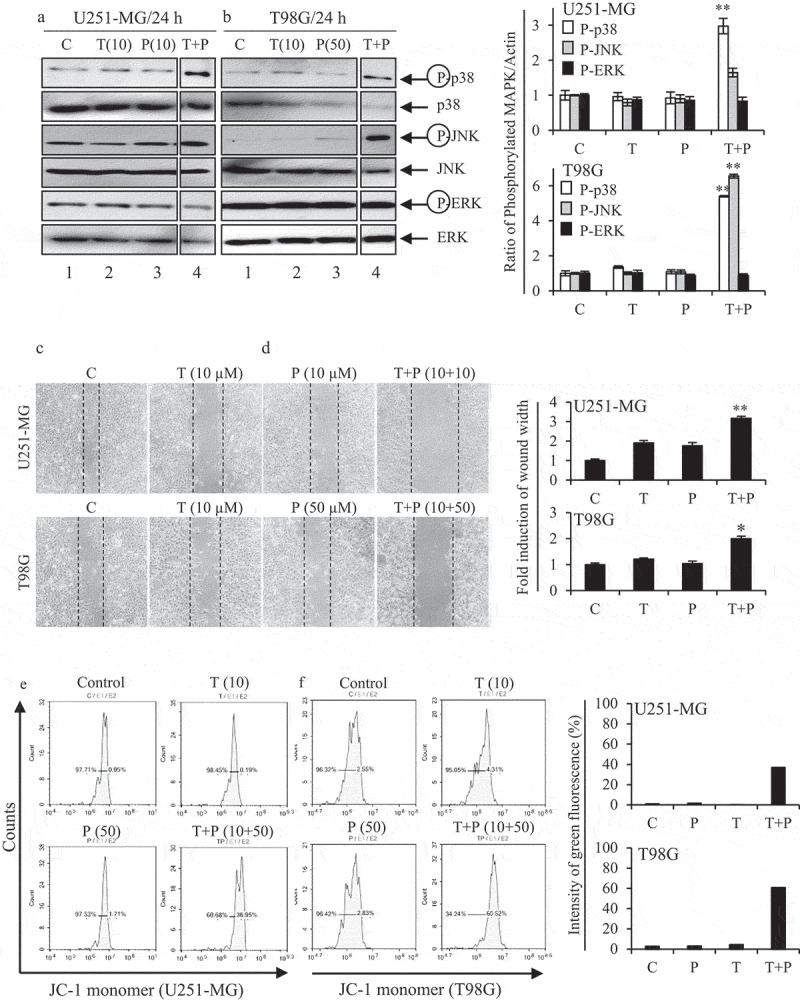
Functional relevance of LowPIP-TMZon GBM cells.

The activation of JNK, p38, and ERK MAPK was tested in both cells after treatment of low concentration PIP, TMZ, or PIP-TMZ. As shown in Figure 4(a,b), low concentration PIP (10/50 µM) and TMZ (10 µM) did not alter phosphorylation of the three MAPKs (*lane 2‒3*) compared to control (*lane 1*). However, low PIP-TMZ (10/50 + 10 µM) showed a visible increase in phosphorylation of JNK and p38 MAPKs (*lane 4*), but not ERK in both cells (Figure 4(a,b)). These experimental outcomes suggested that low PIP/TMZ-mediated apoptosis in GBM cells was associated with the activation of JNK and p38 MAPK. A wound-healing assay was performed to evaluate the effects of cells migration after low PIP-TMZtreatment on GBM cells.

U251-MG (a) and T98G (b) cells were incubated for 24 h in the presence of low concentration PIP and TMZ alone or in combination. Cells were then lysed and total protein was analyzed by western blot. Membranes were probed with each MAPK Ab. Relative expression with respect to total MAPK expression was determined by densitometric analysis and was normalized to the control. Data shown are mean ± SD of three similar experiments (*p < 0.05 and **p < 0.01 as compared with the control group). U251-MG (c) and T98G (d) cells were incubated for 24 h in the presence of low concentration PIP (10/50 µM) and TMZ (10 µM), alone or in combination, and photographed at 24 h by a phase-contrast microscope at 100× magnification. Quantitative data were represented as wound width. Values are expressed as means ± SEM of three independent experiments, *p < 0.05 and **p < 0.01 as compared to the control. U251-MG (e) cells were treated control, low PIP (10 µM) and low TMZ (10 µM), in combination for 24 h. T98G (f) cells were treated control, low concentration PIP (50 µM), and low concentration TMZ (10 µM), in combination for 24 h. Histogram profiles of JC-1 monomer (green fluorescence) were detected using flow cytometry.

Results of the wound-healing assay suggested that treatment with low concentration PIP and TMZ alone did not significantly suppress cells migration, while the low PIP-TMZ treatment increased the suppression effects (Figure 4(c,d)). Low PIP/TMZ-treated cells displayed an approximately 2- to 3-fold wider wound width after 24 h, as compared to the alone cells. These results reveal that co-treatment with low concentration PIP decreased cell motility of the TMZ-resistant GBM cells, which suggests the synergistic anti-proliferative and anti-migration property of low PIP-TMZ.

Mitochondrial membrane depolarization (MMP, *ΔΨ*m) was observed when the cells were treated with PIP, TMZ, or low PIP-TMZ and stained with JC-1 dye. Results of flow cytometry show the loss of *ΔΨ*min in GBM cells which was evident by the increase in the green fluorescence by JC-1 dye staining after low PIP-TMZ treatment (Figure 4(e,f)). The enhancement of green fluorescence in U251-MG and T98G cells was found to be approximately 36.95% and 60.52% for low PIP-TMZ, respectively, in combination (10 + 10/50 + 10 µM). The value of green fluorescence in PIP and TMZ-treated cells was 0.19%/1.71% in U251-MG and 4.31%/2.83% in T98G cells. These results suggest that the low PIP-TMZ treatment significantly reduces MMP of glioma cells and thus induces cellular apoptosis through MMP.

## Discussion

In this study, we investigated whether or not PIP has anti-cancer activity against TMZ-resistant GBM cell lines. Our results showed that PIP could enhance the TMZ sensitization of TMZ-resistant GBM cells. In addition, we could observe that the combination of PIP and TMZ showed synergistic inhibition of cell growth compared to treatments with either alone, suggesting the possibility of avoiding high doses of TMZ clinically.

Induction of apoptosis is considered to be a valuable process in the treatment of various cancers [16]. In the present study, Annexin V-FITC/PI staining showed that the low PIP-TMZ treatment could synergistically enhance the apoptotic death of the TMZ-resistant glioma cells. Recently, a study by Jafri et al. (2019) has reported the cytotoxic effects of PIP against human HeLa cells [15]. The result of this cytotoxic cell death was found to be in agreement with the present study of PIP. However, the ratio of early and late apoptosis of low PIP-TMZ treatment in U251-MG and T98G cells was the opposite. Perhaps it may be because the cancer origin of each GBM cell lines is different. The apoptotic potential of low PIP-TMZ was further tested to verify caspase-8/-9/-3 activation. Our results showed that low PIP-TMZ significantly increased active caspases-8/-9/-3 expression, suggesting that apoptosis induction by the low PIP-TMZ treatment occurred by modulating active caspases-mediated signaling in TMZ-resistant GBM cells.

The induction of apoptosis as well as cell cycle arrest are the major targets for cancer chemotherapy [17]. During cell division cycle, CDK4/6−cyclin D and CDK2−cyclin-E complexes mediate sequential phosphorylation of Rb and allows the G1/S transition [17]. Recent studies have reported that the PIP arrested the cell cycle in G2/M phase and induced apoptosis in HeLa cells [15]. The present flow cytometry results indicated a significant induction of cells in the G2/M phase of the cell division cycle, whereas the S phase population was dramatically decreased. This prompted us to investigate in further detail the mechanism of action of low concentration PIP-TMZ on the cell cycle machinery. RT-PCR of positive cell cycle regulators showed significant inhibition of CDK4/6−cyclin D and CDK2−cyclin-E complexes expression levels upon low concentration PIP-TMZ treatment, suggesting that G1 to S arrest occurs. This may be a possible explanation for the increase in the G1/S ratio.

It is well known that JNK/p38 MAPK pathway is critical for glioma development and progression by regulating several physiological processes, including apoptosis [18,19]. Our study revealed that low PIP-TMZ treatment resulted in increased phosphorylation of JNK and p38 MAPK in U251-MG and T98G cells, implying that low concentration PIP-TMZ combination could induce cell death through the JNK/p38 MAPK-mediated intrinsic apoptotic pathway in GBM cells. Tumor migration ability is an important biological characteristic of malignant tumors [20]. We demonstrated that low PIP-TMZ treatment inhibited cellular migration of glioma cells. On the other hand, low concentration PIP and TMZ alone were not able to inhibit cell migration of glioma cells. There are currently no reports in the literature describing the biological effects of low PIP-TMZ in combination on cancer processes such as migration. Thus, our result suggests that the migration-inhibiting potential may be part of the general anti-cancer mechanism of low PIP-TMZ in tumorigenesis. To better understand the underlying mechanisms, we evaluated the influence of low PIP-TMZ treatment on the MMP (Δ*ψ*m) of glioma cells. An increase in the green fluorescence of JC-1 dye upon low PIP-TMZ treatment indicates a loss of Δ*ψ*m, which is reported to increase in cells undergoing apoptosis [21]. Here, a decrease of Δ*ψ*m is shown in low PIP-TMZ-treated glioma cells for 24 h. Thus, we can propose the following scenario: upon the damage to MMP, cytochrome *c* would be released from the mitochondria into the cytoplasm, leading to activation of caspase-3/-9, thus initiating the mitochondrial-mediated apoptosis.

In summary, our study has revealed that low PIP-TMZ treatment inhibits the proliferation of glioma cells by reducing cell viability, inducing apoptosis and activation of caspase-8/-9/-3, activation of JNK/p38 MAPKs, inhibiting wound healing, and MMP depolarization. In a previous study, Shoba et al. (1998) reported that PIP enhances the serum concentrations, extent of absorption, and bioavailability of curcumin *in vivo* [22]. Thus, it is assumed that the anti-cancer effect of low PIP-TMZ in combination is due to the enhancement of TMZ bioavailability by PIP action. Further studies will be needed to determine the underlying anti-cancer action of low PIP-TMZ combination at *in vivo* level.
